# Modulation of Conflict Processing by Theta-Range tACS over the Dorsolateral Prefrontal Cortex

**DOI:** 10.1155/2019/6747049

**Published:** 2019-07-08

**Authors:** Albert Lehr, Niklas Henneberg, Tarana Nigam, Walter Paulus, Andrea Antal

**Affiliations:** Department of Clinical Neurophysiology, University Medical Center Göttingen, Göttingen 37073, Germany

## Abstract

Behavioral response conflict arises in the color-word Stroop task and triggers the cognitive control network. Midfrontal theta-band oscillations correlate with adaptive control mechanisms during and after conflict resolution. In order to prove causality, in two experiments, we applied transcranial alternating current stimulation (tACS) at 6 Hz to the dorsolateral prefrontal cortex (DLPFC) during Stroop task performance. Sham stimulation served as a control in both experiments; 9.7 Hz tACS served as a nonharmonic alpha band control in the second experiment. We employed generalized linear mixed models for analysis of behavioral data. Accuracy remained unchanged by any type of active stimulation. Over both experiments, the Stroop effect (response time difference between congruent and incongruent trials) was reduced by 6 Hz stimulation as compared to sham, mainly in trials without prior conflict adaptation. Alpha tACS did not modify the Stroop effect. Theta tACS can both reduce the Stroop effect and modulate adaptive mechanisms of the cognitive control network, suggesting midfrontal theta oscillations as causally involved in cognitive control.

## 1. Introduction

In the face of conflicting information, human beings are capable of adjusting their executive control to resolve conflict and perform the appropriate behavior.

During this process, the cognitive control network first detects conflict, then selects and monitors behaviors for attaining a goal. Multiple brain regions jointly exercise inhibitory control when task demands are high to override stimulus-driven behavior. Generally, cognitive control is measured by performance in conflict tasks, like the Stroop task, in which conflicting task-irrelevant information has to be suppressed for responding correctly [1, 2].

In the Stroop color-word task (SCWT), participants indicate the ink color of a color-word while not responding to its semantic meaning. Responses are faster when the semantic meaning and ink color match (congruent, low-conflict, e.g., “Blue” in blue ink) compared to a mismatch (incongruent, high-conflict, e.g., “Blue” in red ink). This response time difference is a function of the congruence and named after its discoverer *Stroop* [3].

Previous electroencephalography (EEG) and functional magnetic resonance imaging (fMRI) studies found that several brain regions are activated during the Stroop task, including the dorsal Anterior Cingulate Cortex (dACC), the dorsolateral prefrontal cortex (DLPFC), and the posterior parietal cortex (PPC) [4–7]. Neuroimaging studies suggest that the left DLPFC is active (300 ms–440 ms) before the dACC (520 ms–680 ms), indicating the left DLPFC as the source of cognitive control implemented for Stroop task performance [4, 6]. Contrarily, the dACC has also been hypothesized to detect conflict at an earlier point in time (220–340 ms) and to engage the DLPFC that then implements cognitive control and resolves the conflict [6, 8]. This apparent contradiction resolves as cognitive control is exerted strongly in trials following an incongruent trial.

Responses in incongruent trials, which are preceded by incongruent trials (iI), are faster than in incongruent trials, which are preceded by congruent trials (cI). Conversely, responses in cI are slower than in cC [5]. This congruency sequence effect (CSE) in trials preceded by incongruent trials is known as the Gratton effect [9]. A conflict in the previous trial recruits greater cognitive control that modulates response times in the subsequent trial. This behavioral adjustment is predicted by the conflict adaptation hypothesis [10–12]. The dACC activity increases in conflict trials [12]. It precedes behavioral adaptations promoted by increased DLPFC activity. Thus, this theory postulates that the interplay of conflict-detecting dACC and allocation of control by the DLPFC is responsible for adaptation of the congruency effect. As conflict trials activate the dACC, engagement of DLPFC reduces both the Stroop effect and the dACC activity in trials after a conflict [5]. If the DLPFC activity is high after engagement, it abolishes the Stroop effect independent of the dACC activation level [6]. Only when the DLPFC activity is low (no earlier engagement by dACC) will the dACC activity correlate with the size of the Stroop effect and negatively with error rates [6]. Therefore, it is conceivable that constant high activation of the DLPFC throughout the Stroop task leads to the abolishment of the Stroop effect. In this study, we aim to increase DLPFC activity exogenously to test this hypothesis.

Transcranial alternating current stimulation (tACS) allows us to causally infer function of oscillatory networks [13]. Through the injection of alternating current into the cortex, membrane potentials of many neurons are rhythmically and simultaneously shifted [14]. This effectively entrains networks exogenously [15]. By controlling the rhythmic brain activity, resulting changes in cognitive functions can be causally attributed to the brain oscillation.

In this study, we have chosen tACS with a frequency of 6 Hz based on previous electrophysiological results. Generally, these electrophysiological studies are in line with and corroborate the findings of neuroimaging studies in the Stroop task. The dACC has been shown to be the generator of mediofrontal negativity in the theta (4–8 Hz) range marked by a stronger negative potential around 450 ms in the incongruent condition [16–18]. This midfrontal theta-band (4–8 Hz) oscillatory activity supposedly reflects neural mechanisms of conflict detection [19].

Furthermore, dACC and left DLPFC couple in the theta phase between conflict detection and resolution [20]. In this phase, dACC activity predicts DLPFC activity, establishing dACC as the driving brain region [21]. This intra-areal theta connectivity is prolonged in incongruent compared with congruent trials [20]. Similar to the Gratton effect for response times, oscillatory power in narrow-band theta (6 Hz–7 Hz) in the left-frontal region is significantly higher in iC trials compared to cC trials, while it is slightly lower in iI trials compared to cI trials [22]. Additionally, non-phase-locked theta power correlates with response times [23]. Thus, the evidence suggests that theta power in the left-frontal region and response times are both influenced by conflict in preceding trials. Natural increase in frontocentral theta power and phase-coupling between dACC and left DLPFC in conflict mediate the increased conflict adaption in the next trial. In the Simon task, the congruency effect was reduced during theta-range tACS directed medially towards the dACC because response times slowed in congruent trials [24]. However, in the color-word Stroop task, the evidence for the importance of dACC and DLPFC interaction for the successful resolution of conflict remains correlational. By stimulating the DLPFC, we aim to illuminate the role of the DLPFC in the cognitive control network during the performance of the Stroop task.

Similarly to our approach, a previous work has also targeted the left DLPFC with a theta-range tACS during decision-making requiring cognitive control [25]. Stimulation increased riskier decision, which confirms the DLFPC as a key region for adaptation of decision strategies. Likewise, theta-range tACS to the left DLPFC increased performance in the easy items of a problem solving test by changing attentional components [26]. The stimulation did however not improve performance in a visual-spatial reasoning task. These results indicate DLPFC specifically as a promising target for low-frequency tACS during cognition, while numerous studies have shown transcranial electrical stimulation to modulate cognitive processes in general [27].

To investigate the efficacy of tACS on conflict processing, we have used the drift diffusion model for conflict tasks (DMC). The DMC is a newly developed extension of the classical drift diffusion model (DDM) [28, 29].

Generally, cognitive processing in conflict tasks is studied by behavioral measures like response time and accuracy, which are influenced by a trade-off between speed and accuracy of response. Cognitive models allow decomposing the response time and accuracy into several parameters underlying the decision process. The DDM models the cognitive processes underlying two-alternative forced choice tasks by assuming that participants start to accumulate for either alternative over the time of the trial. The accumulation of evidence begins at the start of the trial, and as soon as it reaches a certain threshold for one alternative, a decision is being made. Due to noisy sensory input, the accumulation is a stochastic process which occasionally results in error trials. Aside from the decision process, the time needed for nondecisional processes is also accounted for.

In DMC, evidence accumulation is the sum of a controlled process (naming of color) and another, automatic process (recognition of semantic meaning). These processes are summed, either leading to (slower) faster responses in (in)congruent trials. The distribution in time of the automatic process is a gamma density function, peaking early during the trial and decaying afterwards. Therefore, the DMC is well suited as it accounts for both the RT distributions and accuracies of conflict tasks as Stroop, Simon, or Eriksen flanker task [30, 31].

We aimed to externally modulate theta power in the left DLPFC and to thereby causally change the function of the cognitive control network. We employed tACS in the theta range (6 Hz) with a high-definition (HD) electrode montage over the left DLPFC in two experiments, in order to entrain the cortical control network [32, 33]. While both experiment stimulations were compared to sham, the second experiment additionally used tACS in the alpha range (9.7 Hz) as a control. This serves as an active control for the possible frequency-unspecific effects of stimulation.

As mentioned above, we employed GLMM and the newly developed DMC to analyse the effects of tACS on response times and accuracy and also the interaction with the congruency effect [29].

We hypothesized that the cortical control network can be exogenously entrained (via the left DLFPC) by theta tACS. This would result in increased theta power during and after conflict resolution. With longer phase-coupling between the dACC and the left DLPFC, all trials would show activation patterns similar to those in the incongruent trial. This would induce higher cognitive control for the next trial, comparable to the iC or iI conditions of the Gratton effect. Therefore, we predicted a reduced Stroop effect in the active condition compared to the controls. We expected trials which are preceded by a congruent trial to be more strongly affected by stimulation (reduced Stroop effect) as normally they show no conflict adaptation mediated by theta phase-coupling. Consequently, in DMC, the influence of the automatic process on the decision-making should be reduced.

## 2. Methods

### 2.1. Participants

The participants consisted of 22 healthy, right-handed, and native German-speaking adult volunteers, who have normal or corrected-to-normal vision and gave their written informed consent to join the study. They were measured in two experimental groups. The first group consisted of 10 participants (8 females, mean age: 24.4 ± 3.8 years); the second group consisted of 12 participants (8 females, mean age: 25 ± 3.7 years). None of the participants reported neurological or psychiatric disorders and drug-dependency or were taking medication acting on the central nervous system prior to or during the experimental sessions. They were informed about the exclusion criteria and possible adverse effects of tACS. The Ethics Committee of the University Medical Center of Göttingen, Germany, approved the study, which was conducted according to the regulations of the 1964 Declaration of Helsinki.

### 2.2. Experimental Protocol

The experiments were double-blinded, placebo-controlled, and executed in a within-subject design. Experiment 1 (*n* = 10) consisted of an active 6 Hz tACS and a sham stimulation session. Experiment 2 (*n* = 12) had an additional active control condition (alpha tACS). Subjects participated in all sessions of a given experiment. The condition order was counterbalanced across participants to minimize learning effects. Between experimental sessions, a duration of at least 48 hours was maintained to diminish possible carry-over effects of stimulation. Before and after each session, participants reported their level of arousal and indicated their subjective experience of the stimulation after the session. The dependent variables in this study were accuracy and response times (RTs). Additionally, the arousal and sleep quality were also reported.

### 2.3. Task

Participants performed a Stroop color-word task (SCWT) [2], which was designed using the PsychoPy toolbox [34]. In the SCWT, the participants have to indicate the color of the font. The stimuli were four German capitalized color-words (Green, Red, Yellow, and Blue) presented with matching or different font colors. The task was designed as a two-alternative forced choice task, meaning that two colors (Green and Red) mapped onto the same one of the two response buttons that the participant had to press manually. Responses were collected by a dedicated response pad (RB-740; Cedrus Corporation, San Pedro, USA) with a time resolution of 2 ms to 3 ms according to the manufacturer. The congruent condition consisted of the matching color-word and font color (e.g., RED written in red). In the incongruent condition, the color-word and the font color were different but also mapped onto different buttons (e.g., RED in yellow). The CIE Lightness Chroma hue device-independent colorimetric space (Commission Internationale de l'éclairage, 1976) was applied. Red (hue = 30), Blue (hue = 280), Green (hue = 140), and Yellow (hue = 100) had the same lightness (*L* = 51) and chroma level (CL = 55%). The gray fixation cross had the same lightness.

**Figure 1:**
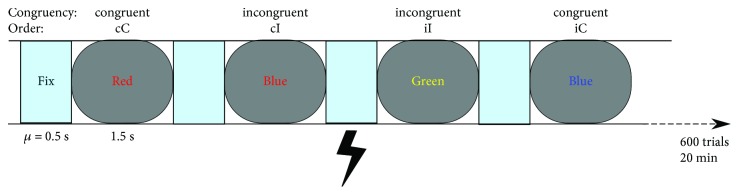
The color-word Stroop task. After practice trials, the participants performed 600 trials within one session while being stimulated by tACS. They responded as quickly and accurately as possible during the 1.5 s of a single trial. Congruent and incongruent trials appeared equally often and were subcategorized depending on the preceding trial.

Each session started with a minimum of 50 practice trials (termination rule: 18 of the last 20 trials correct), and the following main phase consisted of 300 congruent and incongruent trials in a randomized order. The length of a trial was 1.5 s; the mean interstimulus interval lasted 0.5 s (Chi-squared distribution, range 0.3 s–0.7 s) during which a gray fixation cross (hue) was shown. The participants were instructed to respond as quickly and accurately as possible. The SCWT lasted for 20 minutes (Figure 1).

### 2.4. Transcranial Alternating Current Stimulation

Stimulation was delivered by a CE-certified neuroConn multichannel stimulator (neuroConn GmbH, Ilmenau, Germany) throughout the main experimental phase [13].

**Figure 2:**
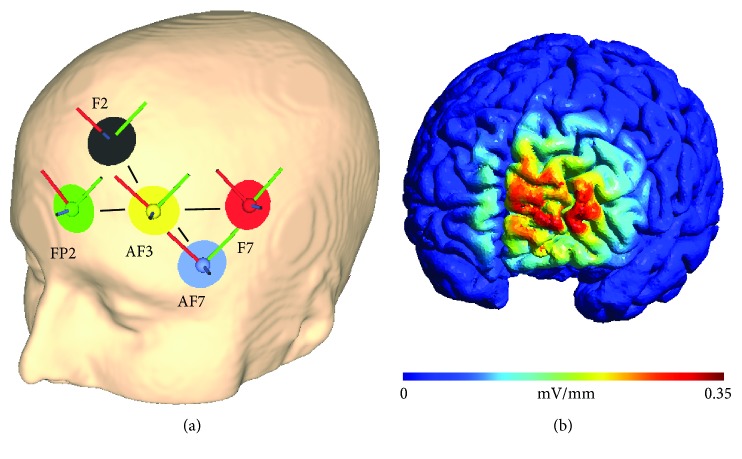
The HD tACS montage for stimulation of the left dorsolateral prefrontal cortex and the modelled electric field strength. (a) The central electrode of the HD montage is centered over AF3. Two pairs of return electrodes form equilateral triangles of 6 cm side length with the central electrode. The distance between both pairs is 10 cm. The return electrodes are located over F5, Fp2, F2, and AF7. (b) The electric field strength is maximal (0.35 mV/mm) over the left prefrontal cortex including the DLPFC. The graphics and electric field strength modelling are derived from SimNIBS 2.0.1.

The high-definition (HD) montage centered over AF3 according to the international 10-10 EEG system with four return electrodes. The return electrodes were positioned over F5, F2, Fp2, and AF7 as in earlier studies targeting the DLPFC [35]. In previous studies, this electrode positioning was used to modulate the activity of the DLPFC. Following the recommendation of previously published modelling studies, the orientation of the plugs and cables was kept constant (facing away perpendicular to the medial line) [36]. Round rubber electrodes of 1 cm radius were fixed on the scalp of participants with the conductive Ten20 paste. This placement leads to left hemispheric frontal stimulation with peak field intensities of 0.3 V/m (Figure 2) according to simulations with the SimNIBS standardized head model [37].

Sinusoidal tACS of 1 mA (peak-to-baseline) intensity and 6 Hz frequency was applied throughout the 20 min duration of the WCST in the active stimulation condition (including 10 s ramp-up and ramp-down periods). Similarly, 9.7 Hz was used as an active control stimulation in the alpha range in the second experiment. Sham stimulation was limited to 30 s (including 10 s ramp-up and ramp-down periods) during the beginning and the end of the SCWT in order to blind the participants while not influencing task performance. The impedances were kept below 15 k*Ω*. The current density at the main electrode was 0.159 mA/cm^2^.

### 2.5. Analysis

The DMC fitting and the organization of behavioral datasets were done in Python. All statistical testing were conducted in R [38].

#### 2.5.1. Generalized Linear Mixed Models

Generalized linear mixed models (GLMMs) are increasingly utilized to analyse complex research designs [39, 40]. They are mainly used for correlated data, e.g., data in which many data points per individual participant exist [41]. This hierarchical structure is analysed without using mean data averaged across the participants' responses. Response time distributions are normally right-skewed, but GLMM does not assume data to be normally distributed [42]. Overall, GLMM allows data to be analysed without reducing it first to mean values [42].

Parsimonious GLMMs were run on nontransformed RTs of correctly answered trials using an identity-linked Inverse Gaussian distribution as recommended by Lo and Andrews [42]. Similarly, for error rates, the GLMM was run including incorrectly answered trials using an identity-linked binomial distribution. We fitted with the packages RePsychLing 0.0.4 [43] and lme4 1.1–15 [44] following recommendations for nongeneralized models [45]. Maximum likelihood was used to fit the GLMM.

The random effects in the final parsimonious model included intercepts for participants and word-color, with slopes of current trial congruency for word-color and within-participant slopes of current trial congruency and stimulation. The random effects account for variance in the data which arises as, for instance, every participant balances the speed-accuracy trade-off differently, which leads to individual response time and accuracy distributions. The categorical two-level fixed effects stimulation (sham, 6 Hz), congruencies of current and preceding trials (both: congruent, incongruent), was sum-coded numerically for the first experiment. In the second experiment, the stimulation (sham, 6 Hz, 9.7 Hz) was also sum-coded numerically, allowing the effect of the active stimulations to be individually compared to sham. Additionally, we could analyse the interaction of the stimulation with the current trial congruency (Stroop effect) and with the current and preceding trial congruencies (Gratton effect). These factorial predictors were contrast-coded to extract their main effects and their interactions on the grand means of reaction time and accuracy. We report the *Z* values and *p* values of the effects via Welch-Satterthwaite's approximation method [46]. All data points are plotted with 95% prediction interval, which marks the range within which the data points would be with a probability of 95% upon resampling.

#### 2.5.2. Fitting Drift Diffusion Models for Conflict Tasks

DMC assumes that the total response time is the sum of the duration of the decision process (*D*) and the residual time (*R*), which includes the sensory processing of stimulus and response execution [29]. Additionally, it assumes that the congruency effect occurs only in the decision process. DMC decomposes the *D* underlying a two-alternative forced choice into several parameters by accounting for the RTs and accuracy of both congruent and incongruent trials. The boundary (*a*) is the threshold which has to be crossed by the evidence accumulation to elicit a decision. The nondecision (Ter) and the variability of the nondecision time (sr) characterize *R*. A controlled process operates on task-relevant information and an automatic process on task-irrelevant information. The controlled process has a constant drift rate (*μ*_c_), whereas the drift rate of the automatic process is changing over time best described by a gamma density function. It decays over time after an early maximum. The amplitude (*ζ*), shape parameter (*α*), and scaling parameter (*τ*) underlie the gamma function [29].

Model fitting was done on individual participants per session (and individual “original” datasets in the recovery study) as described in [29] following these steps:

1. Plausible starting values from the pilot study were drawn for all parameters from a uniform distribution
2. Minimization of *G* ^2^ statistic as a goodness of fit of parameters to the RT distribution and accuracy was done by the Nelder-Mead simplex method [47]. The maximum number of iterations was 250, each with a sample size of 50,000 observations per congruency condition. The integration constant (delta *t* = 1 ms) and diffusion coefficient (sigma = 4) were as in [29]
3. The first two steps were repeated 30 times. Computations were done in parallel with the Göttingen Campus High-Performance Computing Centre as each repetition had a run time of around 30 h

We further analysed the parameters which best fit the data as indicated by the *G* ^2^ statistic. Parameters were statistically compared to infer which parameters had been influenced by the stimulation using permutation tests. The above-mentioned DMC parameters were the dependent variables with the stimulation condition being the independent variable. However, due to poor recovery, the shape and the time characteristic of the automatic process gamma function were excluded from this analysis (see supplementary Figure S1). Permutation tests are nonparametric tests. In the first experiment with its two stimulation conditions, approximative Monte Carlo Fisher-Pitman permutation tests were run for each analysed DMC parameter. As the second experiment included three stimulation conditions, we performed approximative multivariate Kruskal-Wallis tests. In both tests, 10,000 iterations were used [48, 49]. We adopted the hypothesis testing threshold according to the Bonferroni-Holm method for multiple testing.

#### 2.5.3. Arousal and Sleep

Arousal levels in the Stroop task correlate with better performance in congruent trials and worse performance in incongruent trials [50]. For the control, participants self-reported their arousal level before and after performing the Stroop task on a scale from 1 (very tired) to 10 (totally awake). Sleep deprivation increases response times in the Stroop task but leaves interference and accuracy unchanged [51]. Participants self-reported quality from 1 (miserable) to 5 (excellent) and duration (in hours) of their previous night's sleep. All indicators of each session were analysed across stimulation conditions using the two-sided nonparametric paired sample Wilcoxon signed rank test in Experiment 1 and the two-sided nonparametric paired sample Kruskal-Wallis test in Experiment 2.

## 3. Results

### 3.1. First Experiment

Overall accuracy was 94.9% (SD 2.3%), and mean RTs were 624.3 ms (SD 54 ms). Within sham stimulation, accuracy was lower and mean RTs prolonged for incongruent trials (94.6%, SD 2.4%; 652.3 ms, SD 59.1 ms) compared to congruent trials (95.9%, SD 2.8%; 604.2 ms, SD 50.6 ms). Equally, in the active stimulation condition, incongruent trials (93.7%, 2.7%; 638.8 ms, 61.9 ms) were more erroneous and slower than congruent ones (95.3%, SD 2.9%; 602.2 ms, 59.1 ms) (see Table 1).

To assess the effect of the stimulation condition, we were interested in the main effect of the stimulation, its interaction with the congruency of the current trial and its effect on the Gratton effect (i.e., the interaction between congruency of the current and the previous trials). Additionally, we expected an interaction between congruency of the current trial and the stimulation conditions when the preceding trial was either congruent or incongruent. Two generalized linear mixed models were conducted: one for error rates including all trials and the other for the nontransformed response times excluding all error and posterror trials (10.4% of all trials; see Table 2).

For accuracy, significant main effects exist for the congruency (congruent, incongruent) of the current trial (CCT; *Z* = 2.801, *p* < 0.01) but not for stimulation (*Z* = 1.875, *p* = 0.06) or the congruency of the preceding trial (CPT; *Z* = 1.491, *p* = 0.13). Overall, participants were less accurate during incongruent trials (*M* = 93.8%, SE = 0.7%) than during congruent trials (*M* = 95.3%, SE = 1.0%, *p* < 0.001). No effects were found for any higher-order interactions, including the interaction factors CCT *x stimulation* (*Z* = 0.139, *p* = 0.88) or *CCT x CPT x stimulation* (*Z* = 0.87, *p* = 0.38). The accuracy is only influenced by the CCT but not by stimulation or CPT.

**Figure 3:**
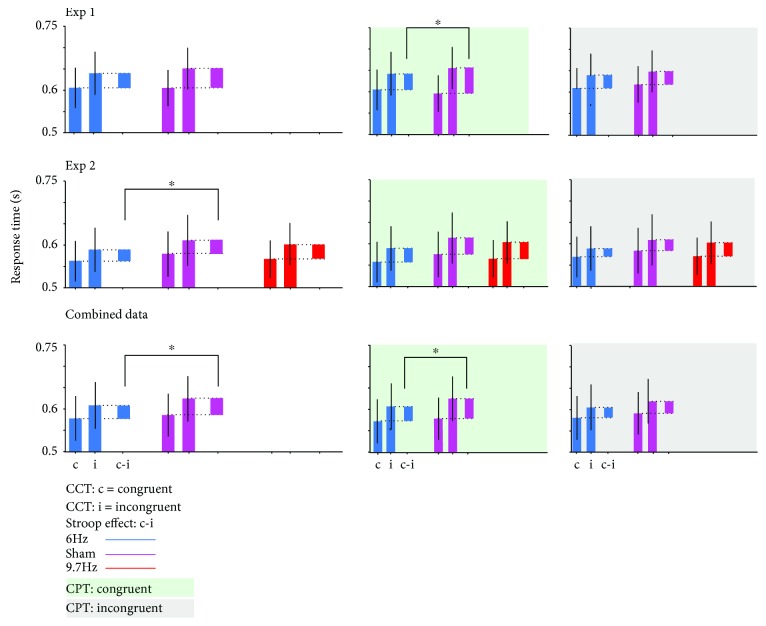
Effect of stimulation on response time. The response times for congruent and incongruent trials and the time difference between these (Stroop effect) are plotted for each stimulation condition individually for all trials in the left panels. In the middle panels, only data of trials which were preceded by a congruent trial are displayed; in the right panels, only for trials preceded by an incongruent trial. Experiment 1 (first row): CCT (size of Stroop effect) and stimulation interact significantly in trials preceded by congruent trials. Experiment 2 (middle row): the interaction CCT x stimulation is significant for all trials but not for the data subsets differentiated by the preceding trial. The active control stimulation in the alpha range did not change the interaction between stimulation and CCT. Combined dataset of both experiments (last row): the interaction between stimulation and CCT is significant across all trials. The significant interaction for trials preceded by congruent trials underlies the effect across all trials. All data is plotted including the 95% confidence interval.

The analysis of the response times revealed significant main effects for CCT (*Z* = 4.37, *p* < 0.001) but neither for CPT (*Z* = 1.06, *p* = 0.28) nor for stimulation (*Z* = 0.49, *p* = 0.61). For CCT, the response times were faster for congruent trials (*M* = 600 ms, SE = 16.1 ms) compared to incongruent (*M* = 639.9 ms, SE = 17.6 ms) trials (Stroop effect). The significant interaction CCT x CPT (*Z* = 3.026, *p* < 0.01) constitutes the Gratton effect, in which the size of the Stroop effect depends on whether the CCT is preceded by a congruent (*M* = 47.9 ms, SE = 7.1 ms) or an incongruent (*M* = 30.9 ms, SE = 4.1 ms) trial. The interaction CCT x stimulation showed a trend (*Z* = 1.847, *p* = 0.06) towards reduced Stroop effect under stimulation (*M* = 33.8 ms, SE = 3.7 ms) compared to sham (*M* = 46.2 ms, SE = 7.1 ms; see Figure 3). The triple interaction CCT x stimulation x CPT narrowly missed the significance criterion (*Z* = 1.828, *p* = 0.06). Further exploration by dividing the dataset according to the congruency of the previous trial revealed a significant interaction CCT x stimulation for trials preceded by a congruent trial (*Z* = 2.87, *p* < 0.01) but no interaction if preceded by an incongruent one (*Z* = 0.01, *p* = 0.98; see Figure 3). Thus, in trials preceded by congruent trials, the stimulation reduces the Stroop effect (*M* = 35.8 ms, SE = 6.1 ms) compared to sham (*M* = 60.2 ms, SE = 11.1 ms).

### 3.2. Second Experiment

Overall accuracy was 97.6% (SD 2.1%), and mean RTs were 578.1 ms (SD 57 ms).

In sham stimulation, accuracy was lower and mean RTs prolonged for incongruent trials (97.8%, SD 1.5%; 604.5 ms, SD 84.9 ms) compared to congruent trials (98.2%, SD 1.7%; 569.5 ms, SD 68.1 ms). Equally, in the 6 Hz condition, incongruent values are 96.9%, SD 3.4%; 583.4 ms, SD 68.6 ms and congruent values are 97.6%, SD 2.5%; 554.9 ms, SD 60.5 ms, and in the active control condition, incongruent values are 97.0%, SD 2.3%; 595.9 ms, SD 69.0 ms and congruent values are 98.1%, SD 1.9%; 560.3 ms, SD 49.2 ms (see Table 1).

To assess the effect of the stimulation condition, we were interested in the main effects of the two active conditions (stimulation: 6 Hz; control: 9.7 Hz). The two interactions were individually compared to sham stimulation. We further investigated their interaction with the congruency of the current trial and their effect on the Gratton effect (i.e., the interaction between congruency of the current and the previous trials). Additionally, we expected a change in their interaction between congruency of the current trial and the stimulation conditions when the preceding trial was either congruent or incongruent. Two generalized linear mixed models were conducted: one for error rates including all trials and the other for the nontransformed response times excluding all error and posterror trials (4.7% of all trials; see Table 3).

For accuracy, significant main effects existed for CCT (*Z* = 2.952, *p* < 0.01) but not for CPT (*Z* = 0.441, *p* = 0.65). Neither the stimulation (*Z* = 0.579, *p* = 0.56) nor the control (*Z* = 0.43, *p* = 0.66) was significantly different from sham. Overall, participants committed more errors during incongruent trials (*M* = 97.1%, SE = 0.6%) than during congruent trials (*M* = 98.0%, SE = 0.5%). For stimulation compared to sham, no effects were found for any higher-order interactions, including the interaction factors CCT *x stimulation* (*Z* = 0.380, *p* = 0.703) and *CCT x CPT x stimulation* (*Z* = 0.876, *p* = 0.38). For the control compared to sham, no effects were found for any higher-order interactions, including the interaction factors CCT *x stimulation* (*Z* = 1.305, *p* = 0.191) and *CCT x CPT x stimulation* (*Z* = 1.572, *p* = 0.11). As in the first experiment, the accuracy is only influenced by the CCT but not by stimulation or CPT.

The analysis of the response times revealed significant main effects for CCT (*Z* = 3.12, *p* = 0.001) and for CPT (*Z* = 2.28, *p* = 0.02). Compared to sham, neither the stimulation (*Z* = 0.78, *p* = 0.43) nor the control (*Z* = 0.06, *p* = 0.95) had an effect on the response times. For CCT, the response times were faster for congruent (*M* = 561.1 ms, SE = 15.0 ms) compared to incongruent (*M* = 592.4 ms, SE = 18.4 ms) trials (Stroop effect). For CPT, the response times were faster when trials were preceded by congruent (*M* = 573.1 ms, SE = 16.5 ms) compared to incongruent (*M* = 580.3 ms, SE = 16.6 ms) trials. The significant interaction CCT x CPT (*Z* = 3.48, *p* < 0.001) constitutes the Gratton effect, in which the size of the Stroop effect depends on whether the CCT is preceded by a congruent (Stroop effect: *M* = 36.7 ms, SE = 7.6 ms) or an incongruent (Stroop effect: *M* = 24.9 ms, SE = 5.0 ms) trial. The active stimulation significantly interacted with CCT (*Z* = 2.11, *p* = 0.03) but not with the interaction CCT x CPT (*Z* = 0.35, *p* = 0.72; see Figure 3).

The size of the Stroop effect depends on whether participants were stimulated with 6 Hz tACS (Stroop effect: *M* = 26.0 ms, SE = 5.0 ms) and the control stimulation (Stroop effect: *M* = 35.0 ms, SE = 8.4 ms) or only sham-stimulated (Stroop effect: *M* = 32.9 ms, SE = 7.2 ms).

The active control did not significantly interact with either CCT (*Z* = 1.44, *p* = 0.14) or interaction CCT x CPT (*Z* = 0.87, *p* = 0.38).

### 3.3. Joint Analysis of Both Datasets

The response time datasets of Experiments 1 and 2 were combined post hoc and reanalysed to increase statistical power (see Table 4). The control condition of Experiment 2 was excluded from further analysis, but the session order was included as a random factor in order to account for increased training effects not balanced out. The analysis of the response times revealed significant main effects for CCT (*Z* = 3.98, *p* < 0.001) but neither for stimulation (*Z* = 1.25, *p* = 0.20) nor for CPT (*Z* = 1.17, *p* = 0.23). For CCT, the response times were faster for congruent (*M* = 579.2 ms, SE = 12.0 ms) compared to incongruent (*M* = 613.3 ms, SE = 14.0 ms) trials (Stroop effect). The significant interaction CCT x CPT (*Z* = 4.40, *p* < 0.001) constitutes the Gratton effect, in which the size of the Stroop effect depends on whether the CCT is preceded by a congruent (Stroop effect: *M* = 41.1 ms, SE = 5.0 ms) or an incongruent (Stroop effect: *M* = 26.3 ms, SE = 3.5 ms) trial. The interaction CCT x stimulation met the significance criterion (*Z* = 2.37, *p* = 0.01), but the triple interaction CCT x stimulation x CPT did not meet the criterion (*Z* = 1.37, *p* = 0.17; see Figure 3). The size of the Stroop effect depended on whether the participants are really stimulated (Stroop effect: *M* = 29.5 ms, SE = 3.2 ms) or sham-stimulated (Stroop effect: *M* = 38.9 ms, SE = 5.1 ms). Further exploration by dividing the dataset according to the congruency of the previous trial revealed a significant interaction CCT x stimulation for trials preceded by a congruent trial (*Z* = 2.65, *p* < 0.01) but no interaction if preceded by an incongruent one (*Z* = 0.71, *p* = 0.47; see Figure 3). Thus, in trials preceded by congruent trials, the stimulation reduces the Stroop effect (*M* = 33.3 ms, SE = 4.3 ms) compared to sham (*M* = 48.9 ms, SE = 7.0 ms).

### 3.4. Diffusion Drift Model for Conflict Task

In Experiment 1, Fisher-Pitman permutation tests investigated statistical differences in the DMC parameter (*a*, Ter, sr, *μ*_c_, *ζ*, *t*_max_, and *t*_90th_) samples recovered for either stimulation conditions. After correcting for multiple comparisons, no statistically significant difference was found for the parameters *a* (*Z* = 0.02, *p* = 1), *μ*_c_ (*Z* = 0.51, *p* = 1), Ter (*Z* = 0.22, *p* = 1), *s*_*t*_ (*Z* = 1.94, *p* = 0.33), *ζ* (*Z* = 0.07, *p* = 1), *t*_90_ (*Z* = ‐0.38, *p* = 0.71), and *t*_max_ (*Z* = ‐0.12, *p* = 1). In Experiment 2, we employed Kruskal-Wallis tests to conduct statistical hypothesis testing on the DMC parameters recovered for all three stimulation conditions. No post hoc tests were performed as there was no statistically significant difference for all parameters after correcting for multiple comparisons: *a* (maxT = 0.41, *p* = 1), *μ*_c_ (maxT = 0.99, *p* = 1), Ter (maxT = 0.38, *p* = 1), *s*_*t*_ (maxT = 1.39, *p* = 1), *ζ* (maxT = 0.93, *p* = 1), *t*_90_ (maxT = 1.07, *p* = 0.71), and *t*_max_ (maxT = 1.18, *p* = 1).

### 3.5. Arousal and Sleep

In the first experiment, the Wilcoxon signed rank test indicated no significant differences between stimulation conditions in mean arousal (*p* = 0.878), sleep quality (*p* = 0.999), and sleep duration (*p* = 0.439). Similarly, neither the Kruskal-Wallis test indicated significant differences between stimulation conditions in mean arousal (*p* = 0.777), sleep quality (*p* = 0.5278), and sleep duration (*p* = 0.935). No subsequent pairwise comparisons were performed.  Table S2 summarizes the descriptive and inferential statistics.

## 4. Discussion

Response conflict increases midfrontal theta dynamics between dACC and the DLPFC [20, 21]. We entrained the cortical control network exogenously by theta tACS during the Stroop task in order to support a causal role of this theta rhythm. For the combined data of both experiments, applying 6 Hz theta tACS reduced the Stroop effect significantly. This effect was driven as expected by reduction in Stroop effect only in trials preceded by congruent trials. The DMC parameters being unchanged did not allow for a more specific characterization.

This is in line with a reduction of congruency effect in the Simon task by theta tACS targeted towards the dACC [24]. This was driven by prolonged response times in congruent trials preceded by congruent trials (cC). Our results and this study provide common evidence for a causal role of mediofrontal theta dynamics in cognitive control.

### 4.1. Electrophysiology of the Stroop Task

EEG recordings in healthy subjects and intracranial recording in epilepsy patients suggest a causal role for the neural oscillatory connection between dACC and DLPFC [20, 21]. The power of theta oscillations is suggested to increase in proportion to the amount of response conflict [20]. Independent of this theta power increase, the phase-coupling in theta range between left DLPFC and dACC changes depending on the congruency of the trial. Specifically, it persists longer and is stronger in incongruent trials [20]. Additionally, DLPFC activity during the Stroop task is associated with activity increase in gamma frequency range (30 Hz–100 Hz), and electrical stimulation in this frequency band led to causal changes in performance [52]. Intracranial EEG recordings revealed for the DLPFC a preparatory period directly after stimulus offset in which theta power increases and gamma oscillations are coupled to theta oscillations [21]. This cross-frequency coupling correlates with accuracy. The detection of conflict at around ~290 ms leads to increases in theta power in dACC, which drives the phase-coupling in theta range with the DLPFC and cross-frequency coupling between DLPFC gamma activity and the phase of the theta activity in dACC. Also, the theta power in dACC between conflict detection and resolution correlates positively with response time. The dACC modulates the DLPFC activity before conflict resolution, whereas the DLPFC modulates the dACC after conflict resolution. The modulation occurs via theta phase synchronization. The gamma power in DLPFC after a response correlates negatively with response times for the next incongruent trial reflecting a preparatory mechanism, and the increased theta phase synchronization is a mechanism for the DLPFC to influence the dACC theta activity. Thus, the directionality of information transfer from DLPFC to dACC via theta phase synchronization and gamma activity cross-frequency coupling (CFC) might implement a different response strategy, which does not require the dACC to be active [21].

We chose the DLPFC as a target since its location at the brain surface allows a more reliable stimulation. Stimulation of the dACC, in which due to its deep location is a more difficult target, has been done however [24, 53, 54]. Active conflict detection and resolution are attributed to dACC activity, whereas the adaptation after response is attributed to DLPFC activity [21]. Increased DLPFC activity after incongruent trials leads to less dACC activity following conflict trials and to reduced congruency effects [6]. Exogenously increased DLPFC activity would reduce dACC activity similarly. Since we targeted the DLPFC with tACS, we hypothesized that the DLPFC-dACC circuitry might have been preferentially influenced when DLPFC was active in adaptation of cognitive control for the next trial. Event-related tACS only during the conflict detection and resolution phase in which the dACC is driving the interaction or only during the adaptation phase could lead to different behavioral outcomes as the latter might have a higher efficacy in manipulating the circuitry's activity. It remains an open question if the DLPFC-dACC circuitry can only be modulated intermittently when stimulating the DLPFC constantly.

It has to be noted that in the first experiment the reduced congruency effect was clearly driven by trials which were preceded by congruent trials. It fits very well in our second hypothesis that stronger cognitive control is exerted when tACS increases the normally low DLPFC activity. However, the Stroop effect was reduced for all trials in the second experiment, not only those preceded by congruent trials. Therefore, the first hypothesis that theta-range tACS reduces the Stroop effect is fulfilled. While 6 Hz tACS reduced the Stroop effect in both experiments, it is a partial replication as different subsets of data are affected. In the combined dataset of the studies, both effects survive the joint analysis, showing a general effect of DLPFC on Stroop effect across all participants. Both experiments were designed equally except for the active control condition. Participants acted as their own control by participating in all sessions of an experiment, which cancels out possible difference in performance between the experiments. Therefore, the pooling of the data of both experiments is statistically valid and allows the interpretation of trends underlying both datasets. Inconsistent effects of tACS have been reported before in internal replications [55], but the results of both experiments in this study causally corroborate the importance of DLPFC activity during the Stroop task.

We confirmed the validity of the used DMC by recovering simulated data (see Supplementary Material), replicating an earlier study [56]. We hypothesized the DMC parameters to reflect increased conflict adaptation and therefore a decreased influence of the automatic process on the stochastic decision process. This would entail a combination of reduced amplitude and reduced *t*_90_ or *t*_max_ of the automatic process (word reading), which we did not find when estimating the parameters [29]. Therefore, the DMC models indicate for both experiments that the influence of the automatic process remained the same for sham and active stimulation. In the first experiment, 6 Hz stimulation reduced the Stroop effect for all trials by 12.4 ms (38.8%) compared to sham; in the second experiment, by 6.9 ms (22.0%). The breakdown of the behavioral data of each participant and session into 7 underlying DMC parameters reduced the statistical power of the subsequent analysis. Therefore, the DMC was insufficient to detect small absolute changes in both response times and accuracy in this study.

The stimulation frequency of 6 Hz chosen as oscillatory power in narrow-band theta (6 Hz–7 Hz) in the left-frontal region correlates with reaction time in conflict adaptation [22]. While most studies documenting the increased phase-coupling between DLPFC and dACC do not further delimit the frequency beyond being in the theta range, it has been suggested that dACC theta phase at 5 Hz modulates gamma activity in the DLPFC [21]. As the frequency of 6 Hz is at the center of the range of individualized theta frequencies in a study employing a Simon task, we are confident that 6 Hz stimulation was an appropriate choice [24]. In particular, in the context of theta-gamma coupling, the option of superimposing more gamma cycles on a longer theta wave may provide better effects in future experiments. Further studies might obtain stronger abolishments of the Stroop effect when stimulating at the individuals' theta peak frequencies [57].

Our choice of active control frequency in the second experiment fell on a nonharmonic frequency in the alpha range. In previous studies, alpha power decreased after conflict trials as it marks higher arousal [22, 58] but has no indicated role in conflict detection or resolution.

Alpha tACS showed a trend towards a reduced congruency effect during the Simon task in an earlier study [24]. Our results do not show this trend, and therefore, the effect of theta tACS on the conflict processing cannot be attributed to unspecific stimulation effects.

### 4.2. Outlook and Clinical Relevance

For future studies, the stimulation of the DLPFC in a broad gamma range would be a promising target as DLPFC gamma power after response predicted response times in subsequent trials. Also, theta-gamma cross-frequency stimulation paradigms promise stronger abolishment of the Stroop effect as they effectively change functionality of distant brain regions which exhibited this type of cross-frequency behavior [57].

It is of note that the theta stimulation to the DLPFC could be equally effective if limited to the time after response. Therefore, the effect of stimulation on adaptation could be isolated while not interfering with conflict detection and resolution. A transfer of the stimulation paradigm to different conflict tasks could show causally if the cognitive control network's physiology is equal in all these tasks.

The Stroop task is a frequently applied neurophysiological test to study neural mechanisms of inhibitory control and its dysfunction [59]. Clinically diverse disorders as chronic alcoholism, schizophrenia, and age-related memory impairment are associated with increased interference in the Stroop task [60–62], which is a biomarker for the inability to correctly inhibit automatic responses and to maintain goal-directed behavior [59]. Both these executive functions are essential to living a well-adapted life, and their restoration is desirable [63].

## 5. Conclusion

This is the first study stimulating the DLPFC by theta tACS. We demonstrate that the cognitive control network can also be influenced by stimulation targeting the DLPFC. We were able to reduce the Stroop effect in a subset of trials over both experiments. The equalization of response times in congruent and incongruent trials suggests that postconflict adaptation was changed. We propose the hypothesis that theta stimulation of the DLPFC is effective in changing preparatory mechanisms after conflict resolution. The key questions to be clarified are whether (a) gamma tACS leads to more reduction of the Stroop effect, (b) theta stimulation applied only after a conflict resolution is equally effective, and (c) the results are generalizable to other conflict tasks.

## Figures and Tables

**Table 1:** Descriptive statistics of both experiments. For both experiments, the difference in behavior between congruent and incongruent trials is broken down per stimulation condition. Mean values are reported with their respective standard deviation.

	Accuracy (%)	Response times (ms)
*Experiment 1*	94.9 ± 2.3	624.3 ± 54
Sham		
Congruent	95.9 ± 2.8	604.2 ± 50.6
Incongruent	94.6 ± 2.4	652.3 ± 59.1
6 Hz		
Congruent	95.3 ± 2.9	602.2 ± 59.1
Incongruent	93.7 ± 2.7	638.8 ± 61.9
*Experiment 2*	97.6 ± 2.1	578.1 ± 57
Sham		
Congruent	98.2 ± 1.7	583.4 ± 68.6
Incongruent	97.8 ± 1.5	604.5 ± 84.9
6 Hz tACS		
Congruent	97.6 ± 2.5	554.9 ± 60.5
Incongruent	96.9 ± 3.4	583.4 ± 68.6
9.7 Hz tACS		
Congruent	98.1 ± 1.9	560.3 ± 49.2
Incongruent	97.0 ± 2.3	595.5 ± 69

**Table 2:** Statistical analysis of the first experiment. The results of the GLMMs are shown for both accuracy and response time data of the first experiment. Additionally, the response times were divided according to the congruency of the previous trial in additional model runs. For every factor, the mean values and standard errors of each factor level are reported. The results of the statistical testing of the difference between these mean values are also reported as *Z* and *p* values.

	Estimate (mean ± SE)	*Z* value	*p* value
*Accuracy (%)*			
CCT	*Δ*1.5	2.801	<0.01
Congruent	95.3 ± 1.0		
Incongruent	93.8 ± 0.7		
CPT	*Δ*0.6	1.491	0.13
Congruent	93.4 ± 1.2		
Incongruent	94.0 ± 1.0		
Stimulation	*Δ*0.9	1.875	0.06
Sham	94.2 ± 1.1		
6 Hz	93.3 ± 1.1		
*Response times (ms)*			
CCT (Stroop effect)	*Δ*39.9	4.37	<0.001
Congruent	600 ± 16.1		
Incongruent	639.9 ± 17.6		
CPT	*Δ*3.2	1.06	0.28
Congruent	624.3 ± 22.9		
Incongruent	627.5 ± 22.9		
Stimulation	*Δ*6	0.49	0.61
Sham	628.9 ± 22.7		
6 Hz	622.9 ± 24.4		
CCT x stimulation	*Δ*12.4	1.847	0.06
Stroop effect (sham)	46.2 ± 7.1		
Stroop effect (6 Hz)	33.8 ± 3.7		
CCT x CPT	*Δ*17	3.026	<0.01
Stroop effect (CPT: congruent)	47.9 ± 7.1		
Stroop effect (CPT: incongruent)	30.9 ± 4.1		
CCT x CPT x stimulation	*Δ*23.1	1.828	0.06
*Response times (ms)–data divided according to congruency of previous trial (CPT)*			
CPT = congruent			
CCT x stimulation	*Δ*24.4	2.87	<0.01
Stroop effect (sham)	60.2 ± 11.1		
Stroop effect (6 Hz)	35.8 ± 6.1		
CPT = incongruent			
CCT x stimulation	*Δ*1.3	0.01	0.98
Stroop effect (sham)	30.9 ± 5.9		
Stroop effect (6 Hz)	32.2 ± 6.3		

SE: standard error.

**Table 3:** Statistical analysis of Experiment 2. The results of the GLMMs are shown for both accuracy and response time data of the second experiment. For every factor, the mean values and standard errors of each factor level are reported. The results of the statistical testing of the difference between these mean values are also reported as *Z* and *p* values.

	Estimate (mean ± SE)	*Z* value	*p* value
*Accuracy (%)*			
CCT	*Δ*0.9	2.952	<0.01
Congruent	98.0 ± 0.5		
Incongruent	97.1 ± 0.6		
CPT	*Δ*0.1	0.441	0.65
Congruent	98.6 ± 0.6		
Incongruent	98.7 ± 0.4		
Stimulation			
Sham	98.5 ± 0.3		
6 Hz (vs. sham)	*Δ*0.2	0.579	0.56
6 Hz	98.3 ± 0.5		
9.7 Hz (vs. sham)	*Δ*0	0.43	0.66
9.7 Hz	98.5 ± 0.4		
*Response times (ms)*			
CCT (Stroop effect)	*Δ*31.3	3.12	0.001
Congruent	561.1 ± 15.0		
Incongruent	592.4 ± 18.4		
CPT	*Δ*7.2	2.28	0.02
Congruent	573.1 ± 16.5		
Incongruent	580.3 ± 16.6		
Stimulation			
Sham	585.1 ± 24.1		
6 Hz (vs. sham)	*Δ*4.6	0.78	0.43
6 Hz	580.5 ± 21.8		
9.7 Hz (vs. sham)	*Δ*2	0.06	0.95
9.7 Hz	584.9 ± 27.5		
CCT x stimulation			
Stroop effect (sham)	32.9 ± 7.2		
Stroop effect (6 Hz vs. sham)	*Δ*6.9	2.11	0.03
Stroop effect (6 Hz)	26.0 ± 5.0		
Stroop effect (9.7 Hz vs. sham)	*Δ*2.1	1.44	0.14
Stroop effect (9.7 Hz)	35.0 ± 8.4		
CCT x CPT	*Δ*11.8	3.48	<0.001
Stroop effect (CPT: congruent)	36.7 ± 7.6		
Stroop effect (CPT: incongruent)	24.9 ± 5.0		
CCT x CPT x stimulation			
CCT x CPT (6 Hz vs. sham)	*Δ*0.7	0.35	0.72
CCT x CPT (9.7 Hz vs. sham)	*Δ*1.8	0.87	0.38

SE: standard error.

**Table 4:** Statistical analysis of combined dataset. The results of the GLMMs are shown for both accuracy and response time data of the combined dataset of both experiments. Additionally, the response times were divided according to the congruency of the previous trial in additional model runs. For every factor, the mean values and standard errors of each factor level are reported. The results of the statistical testing of the difference between these mean values are also reported as *Z* and *p* values.

	Estimate (mean ± SE)	*Z* value	*p* value
*Response times (ms)*			
CCT (Stroop effect)	*Δ*34.1	3.98	<0.001
Congruent	579.2 ± 12.0		
Incongruent	613.3 ± 14.0		
CPT	*Δ*3.7	1.17	0.23
Congruent	595.1 ± 25.5		
Incongruent	598.8 ± 25.5		
Stimulation	*Δ*12.3	1.25	0.20
Sham	603.1 ± 20.3		
6 Hz	590.8 ± 19.3		
CCT x stimulation	*Δ*9.4	2.37	0.01
Stroop effect (sham)	38.9 ± 5.1		
Stroop effect (6 Hz)	29.5 ± 3.2		
CCT x CPT	*Δ*14.8	4.40	<0.001
Stroop effect (CPT: congruent)	41.1 ± 5.0		
Stroop effect (CPT: incongruent)	26.3 ± 3.5		
CCT x CPT x stimulation	*Δ*13.3	*1.37*	*0.17*
*Response times (ms)—data divided according to congruency of previous trial (CPT)*			
CPT = congruent			
CCT x stimulation	*Δ*15.6	2.65	<0.01
Stroop effect (sham)	48.9 ± 7.0		
Stroop effect (6 Hz)	33.3 ± 4.3		
CPT = incongruent			
CCT x stimulation	*Δ*2.3	0.71	0.47
Stroop effect (sham)	27.9 ± 5.7		
Stroop effect (6 Hz)	25.6 ± 3.9		

SE: standard error.

## Data Availability

The behavioral data used to support the findings of this study are available from the corresponding author upon request.

## Supplementary Materials

Supplementary Materials The supplementary material is a recovery study for one of the used models, which shows that the model works and can be applied to the data. Additionally, it includes one table specifying the age and gender of the participants and one table showing the quality of sleep and the arousal parameters.
