# Supplementary material for: Modulation of Conflict Processing by Theta-Range tACS over the Dorsolateral Prefrontal Cortex
Source: Neural Plast. 2019 Jul 8;2019:6747049. doi: 10.1155/2019/6747049 (PMC6644240; doi:10.1155/2019/6747049)
Supplement: Supplementary Materials — The supplementary material is a recovery study for one of the used models, which shows that the model works and can be applied to the data. Additionally, it includes one table specifying the age and gender of the participants and one table showing the quality of sleep and the arousal parameters. [file 6747049.f1.pdf]

Supplementary Material

Diffusion drift model for conflict task

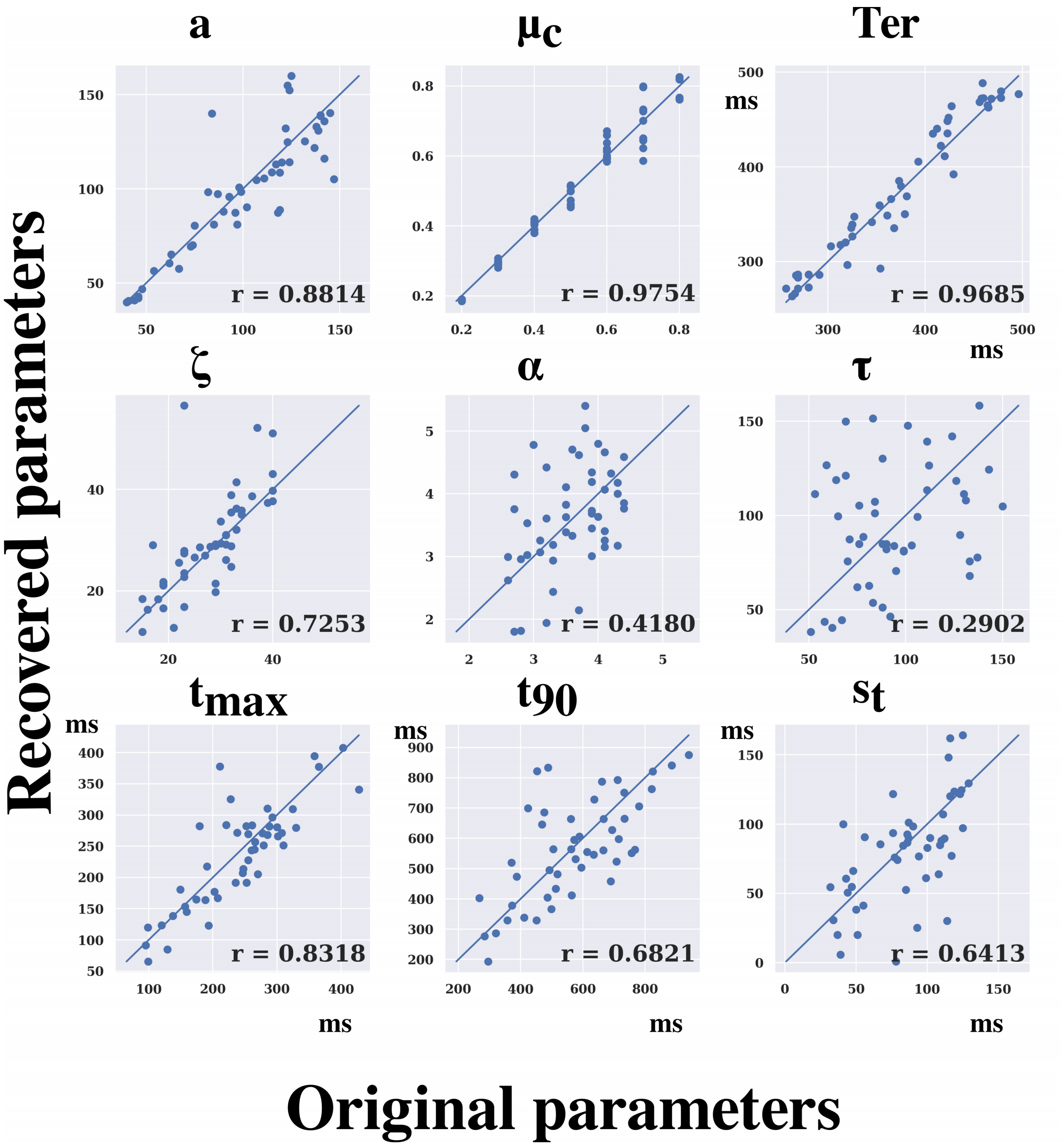

Figure S1: Correlation coefficients between the original and recovered parameters of the recovery study. *Values close to the identity line indicate well-recovered parameter values. The parameters a (Boundary of decision process);* μ_c *(drift rate of controlled process); Ter (Non-decision time); ζ (peak amplitude), α (shape) and τ (characteristic time) for the automatic activation gamma function;* t_{max} *(latency of the peak amplitude),* t_{90} *(0.9 quantile of automatic process gamma function) of the automatic process gamma function;* s_t *(variability of the non-decision time) are shown.*

Recovery study for DMC

DMC has been methodologically tested in a parameter-recovery study by [52]. To the best of our knowledge DMC has not been used with Stroop task data. We defined starting values for fitting based on pilot study results and conducted a similar parameter-recovery study for 48 data sets of both 300 simulated congruent and incongruent trials. In the recovery study the average G^2 statistic across all 48 datasets was 6.9, indicating good model fits. In the first experiment the average G^2 statistic was 23.2, in the second experiment, 18.6.

Data Recovery

Similar to [52], we sampled the value of each DMC parameter from uniform distributions. The ‘original data’ was created with these underlying parameters, which were supposed to be recovered using the DMC. As the original parameter values are known, the quality of the DMC can be assessed by calculating the correlation coefficients r between original and recovered data. For each parameter the recovery was judged to be recovered either poorly ($r < 0.5$), fairly ($0.5 < r < 0.75$), well ($0.75 < r < 0.9$) or excellently ($r > 0.9$).As shown in Figure S1, two parameters were recovered excellently, the drift rate of the controlled process (μ_c, r = 0.9754) and the non-decision time (Ter, r = 0.9685). The boundary of the decision process (a, r = 0.8814) and latency of the peak amplitude of the automatic process (t_{max}, defined as $\tau(\alpha-1)$, r = 0.8318) were well recovered, the variability of the non-decision time (s_t, r = 0.6413), the amplitude of the automatic process (ζ, r = 0.7253) and the 0.9 quantile of the automatic process gamma function (t_{90}, r = 0.6821) fairly. The shape (α, r = 0.4180) and the characteristic time (τ, 0.2902) of the describing the automatic process gamma function were excluded from further analysis as they are incoperated in t_{max} and t_{90} and as recovery was poor similar to the earlier recovery study[52]. The seven parameters with fair or better recovery were used in a linear model to investigate changes in parameters induced by electrical stimulation.

The age, gender, arousal and sleep of participants

Table S1: Gender and age of participants in both experiments.

	n	Mean age (± SD)
Experiment 1		24.4 ± 3.8 years
Women	8	
Men	2	
Experiment 2		25 ± 3.7 years
Women	8	
Men	2	

Table S2: Arousal and Sleep indicators. *The non-parametric paired samples Wilcoxon signed rank test was used for hypothesis testing in Experiment 1, the non-parametric paired sample Kruskal-Wallis test in Experiment 2.*

	**Condition**	**Mean ± SD**	**Test (p-value)**
**Mean Arousal (1 – 10; max: 10)**			
**Experiment 1**			
	6 Hz	5.6 ± 2.3	0.878
	Sham	5.85 ± 1.5	
**Experiment 2**			
	6 Hz	6.3 ± 1.8	0.777
	9.7 Hz	6.6 ± 1.5	
	Sham	6.2 ± 1.5	
**Sleep quality (1 – 5; best: 5)**			
**Experiment 1**			
	6 Hz	3.9 ± 0.7	0.999
	Sham	3.8 ± 0.9	
**Experiment 2**			
	6 Hz	4.0 ± 0.9	0.5278
	9.7 Hz	4.0 ± 0.9	
	Sham	3.6 ± 1.0	
**Sleep duration (in h)**			
**Experiment 1**			
	6 Hz	7.3 ± 0.6	0.439
	Sham	6.8 ± 1.4	
**Experiment 2**			
	6 Hz	7.2 ± 1.4	0.935
	9.7 Hz	7.3 ± 1.5	
	Sham	6.9 ± 1.7	
